# Prevalence of Frailty in the Middle East: Systematic Review and Meta-Analysis

**DOI:** 10.3390/healthcare10010108

**Published:** 2022-01-06

**Authors:** Bader A. Alqahtani, Mohammed M. Alshehri, Ragab K. Elnaggar, Saad M. Alsaad, Ahmed A. Alsayer, Noura Almadani, Ahmed Alhowimel, Mohammed Alqahtani, Aqeel M. Alenazi

**Affiliations:** 1 Department of Health and Rehabilitation Sciences, Prince Sattam Bin Abdulaziz University, Al-Kharj 11942, Saudi Arabia; ragabelnaggar@gmail.com (R.K.E.); a.alhowimel@psau.edu.sa (A.A.); aalenazi@kumc.edu (A.M.A.); 2 Physical Therapy Department, Jazan University, Jazan 82412, Saudi Arabia; phdalshehri@gmail.com; 3 Medical Research Center, Jazan University, Jazan 45142, Saudi Arabia; 4 Department of Physical Therapy, Faculty of Physical Therapy, Cairo University, Giza 12613, Egypt; 5 Department of Family and Community Medicine, College of Medicine, King Saud University, Riyadh 11451, Saudi Arabia; salsaad@ksu.edu.sa; 6 College of Science and Arts, Taibah University, Medina 42353, Saudi Arabia; AlsayerAh@gmail.com; 7 Community Health Nursing Department, Nursing College, Princess Nourah Bint Abdulrahman University, Riyadh 84428, Saudi Arabia; naalmadani@pnu.edu.sa; 8 Royal Saudi Land Forces, Medical Services, Riyadh 13213, Saudi Arabia; manq1@gmail.com

**Keywords:** frailty, frailty index, Middle East, prevalence

## Abstract

(1) We aimed to systematically search available data on the prevalence of frailty among community-dwelling elders in Middle Eastern countries. The results from available studies are cumulated to provide comprehensive evidence for the prevalence of frailty. (2) Methods: A meta-analysis was done. A literature search was carried out using PRISMA guidelines in PubMed, Web of Science, and SCOPUS websites for studies up to 2020. Inclusion criteria entailed all primary studies conducted in Middle Eastern countries on frailty in community-dwelling older adults aged 60 years and older. (3) Results: A total of 10 studies were selected for this study. Random-effects meta-analysis of nine studies indicated there was a pooled prevalence rate of 0.3924 with a standard error of 0.037. This pooled prevalence point estimate of 0.3924 was statistically significant (*p* < 0.001). The Egger’s regression test and the trim-fill method for detecting publication bias did not detect any evidence of publication bias in the sample of included studies. The Egger’s regression test was not statistically significant. The trim-fill method indicated zero studies were missing on either side; (4) Conclusions: The study’s findings indicate that the prevalence of frailty is higher in Middle Eastern nations. Despite indications that many of these nations’ populations are rapidly ageing, we presently lack information on the incidence of frailty in these populations; this information is essential to health, policymakers, and social care planning.

## 1. Introduction

Over the past two decades, there has been a growing interest in frailty among elderly adults to identify those most in need of medical attention and healthcare services [1]. Despite recent initiatives, it was, however, difficult to reach a consensus on what constitutes frailty [2]. There is a common agreement that frailty is a state of increased vulnerability that results from ageing-related cumulative decline across multiple physiological systems, which compromise the ability to cope with every day or acute stressors and increase the risk of developing a dependency, morbidity, and/or mortality [3,4]. In the absence of a gold standard, Fried and colleagues [5] have described frailty as a condition that meets three out of the five phenotypic criteria indicatives of reduced energetics, specifically, low physical activity, slow walking speed, low energy, low grip strength, and unintentional weight loss. Otherwise, frailty has been conceptualized as a risk index by counting the time-accumulated deficits known as frailty index (FI), which include geriatric syndromes (i.e., delirium, falls, and urinary incontinence), physical and cognitive impairments, diseases, disability, and psychosocial risk factors [6].

Even though it is more appealing for use in clinical settings, the rules-based Fried model has been criticized as being more dependent on physical dimensions with little attention paid to mental wellbeing and comorbidities [2]. On the other side, the FI is informative and is likely more sensitive for predicting the adverse health outcomes and identifying the health status trajectories over time due to its delicate graded risk scale and its robust clinical inferences concerning the number and actual composition of its items (typically 30–40 items) [7]. While the practical implementation of these two most frequently used determinants of frailty remains arguable [8], the analysis and discussion in this study focus on subgroups representative of the Fried phenotype- and FI-based estimates of frailty prevalence among community-dwelling elders in the Middle East countries to provide a shred of all-inclusive evidence.

Longitudinal studies have found numerous negative outcomes linked to frailty, which could have a direct effect on the quality of human lives and society in general. These involve falling, mobility deterioration, injury, hospitalization, and increased mortality risk [5,9,10,11]. Assessing frailty in all Middle East countries can be even more complicated, maybe because, in some regions, the health resources are scarce, lack of supplies, lack of qualified personnel, or lack of suitable validated assessment instruments, which may influence the assessment of frailty. Depending on the adopted operational concept of frailty and the characteristics of the population surveyed, the global prevalence of frailty among the elderly population varies between 4% and 59.1% [12]. According to a recent study on the prevalence of frailty among community-dwelling older adults based on the income levels of nations, the pooled prevalence of frailty was 12.3% and pre-frailty was 55.3% among the middle-income countries which were high as compared to high-income countries in which the prevalence estimate of frailty was 8.2% and pre-frailty was 43.9% [13]. Studies conducted in Europe, the United States, Canada, and Australia have shown that frailty rates ranged between 4 and 60% [14]. The available data from the Middle East countries revealed an overall prevalence of frailty among individuals who are 60 years old or more of 47% in the United Arab Emirates [15], 40% in Saudi Arabia [16], 66.3% in Egypt [17], 60% in Iran [18], 28.7% in Turkey [19], and 81.3% in Lebanon [20]. However, these discrepancies in the prevalence of frailty require a comprehensive overview of frailty prevalence in these countries since there are commonly shared geographical and demographical factors.

Despite evidence that populations are rapidly ageing, there are no up-to-date studies that collated all the epidemiological data available from Middle Eastern countries, which could be an urgent need for identification and execution of successful long-term care policies that address the diverse needs for frail older adults in these countries, thus promoting healthier ageing and decreasing the impact of frailty on the health care systems. Therefore, this review and meta-analysis study intended to systematically search available data on the prevalence of frailty and pre-frailty among community-dwelling elders in Middle Eastern countries and pool the results from available studies to provide ample evidence for the prevalence of frailty.

## 2. Materials and Methods

### 2.1. Protocol

The systematic review protocol was registered in PROSPERO (CRD42021226807). The presentation of the results from this systematic review and meta-analysis follows the PRISMA guidelines [21].

### 2.2. Eligibility Criteria

Eligible studies for this review met the following criteria: (1) cross-sectional studies that have measured frailty in community-dwelling older adults using well-validated frailty tools (2) conducted in Middle Eastern countries on people aged 60 years or older. In addition, we excluded any study that has examined frailty in older adults with a specific disease or condition, included participants from outside Middle Eastern countries or in a non-English language.

### 2.3. Search Strategy

A computerized systematic literature search of PubMed, Web of Science, and SCOPUS carried out from June 2020 to December 2020 was performed, without publication year limits to capture all 103 possible relevant titles. The keywords and phrases (in different combinations) include frailty, the prevalence of frailty, frailty incidence, frailty epidemiology, frail elderly, frail, middle east countries (Saudi Arabia, Iran, Turkey, Jordan, Qatar, United Arab Emirates, Bahrain, Kuwait, Oman, Yemen, Iraq, Israel, Palestine, Cyprus, Syria, Lebanon, Egypt, Sudan, and Libya). These keywords were linked using Boolean operators and searched individually on the chosen databases. The Boolean operators used are AND, OR, and NOT. For instance, the first search was “frailty AND old age”. The same procedure was repeated using the NOT and OR, including adjusting the keywords and the operators in different forms to obtain search results.

### 2.4. Study Selection

Studies were evaluated for meeting the eligibility criteria. All studies conducted in Middle Eastern countries that examined the prevalence of frailty were selected for this review. The main reviewed outcome was the frailty prevalence rate. Screening of eligible studies was conducted by two of the authors (BA and AA). Disagreements regarding eligibility were resolved by a third reviewer (MA).

### 2.5. Data Extraction and Quality Assessment

A double coding approach was implemented to extract data. Two of the investigators (BA and AA) extracted data by using a standardized data extraction form. Extracted data included authors, year of publication, study country, sample size, male percentage, age, outcomes, and overall prevalence. Any disagreement was discussed until a consensus is reached. The quality of included studies was assessed using an adapted version for cross-sectional studies of the Newcastle-Ottawa Quality Assessment Scale [22]. The scale examined three areas including study group selection, group comparability, and outcome of interest. The final maximum score is 9 stars for the nonrandomized studies. The quality of included studies was categorized as follows: very good quality for 9–10 scores, good for 7–8 scores, satisfactory for 5–6 scores, and unsatisfactory for score 0–4. A consensus between reviewers was reached in the case of any scoring discrepancies.

### 2.6. Data Synthesis and Analysis

A random-effects meta-analysis and 95% CIs was used to estimate the pooled prevalence rate using R software with metaphor and tidyverse packages. Potential publication bias across included studies was graphically examined using funnel plots, and asymmetry in the funnel plots was evaluated using the Egger’s test.

## 3. Results

**Figure 1:**
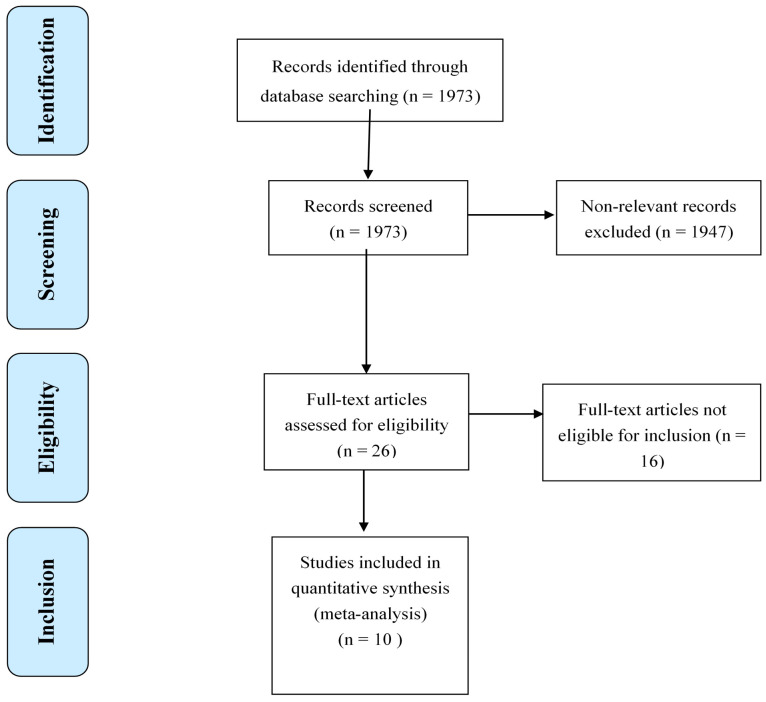
Flowchart of the systematic review and meta-analysis according to the PRISMA guidelines.

The literature search resulted in a total of 1973 studies (PubMed: 1346, Web of Science: 508, and SCOPUS: 119), of which the full text of 26 studies were reviewed and only 10 studies were eligible to be included in the meta-analyses. The sixteen studies were excluded because they did not meet the criteria of age limit (60 years and older), and the lack of clear frailty definition. Figure 1 shows the complete selection process for the included studies.

The total number of participants included in this review was 5666 ranging from 47 to 1200. The overall mean age was 71.1 ± 3.2 years for all included studies. Only six countries have reported the prevalence of frailty including Saudi Arabia, Turkey, Lebanon, Egypt, Iran, and the United Arab Emirates (UAE). The total prevalence rate ranged from 4.41 to 81.3 (Table 1).

The quality of all of the included studies ranged from 3 to 8 as per the Newcastle-Ottawa scale with a mean score of 6.18 for all included studies indicating satisfactory quality. One study scored 3 [17] two studies scored 5 [16,27] two studies scored 6 [23,24] five studies scored 7 [15,19,25,26] and one study scored 8 [20] No studies scored 1, 2, 4 or 9. Table 2 shows the quality rating for all included studies.

**Figure 2:**
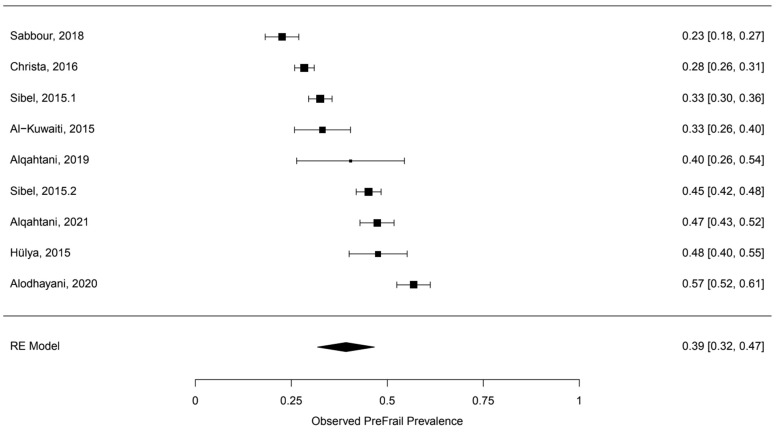
Forest plot of the observed pre-frail prevalence.

A random-effects meta-analysis of nine studies indicated there was a pooled prevalence rate of 39.24% with a standard error of 0.037 for pre-frail. This pooled prevalence point estimate of 39.24% was statistically significant (*p* < 0.001) with a 95% confidence interval of [31.82, 46.66]. Figure 2 presents the results of the meta-analysis along with upper and lower 95% confidence interval bounds for the pooled prevalence estimates. The test for heterogeneity was found to be significant, Q (df = 8) = 227.0042, *p* < 0.001. For I2, the I2 of this meta-analysis was found to be 96.32, which indicated a high heterogeneity between the studies. The Egger’s regression test and the trim-fill method for detecting publication bias did not detect any evidence of publication bias in the sample of included studies. The results for Egger’s regression test were not statistically significant (z = 0.3629, *p* = 0.7167). The trim-fill method indicated zero studies were missing on either side (Figure 2).

**Figure 3:**
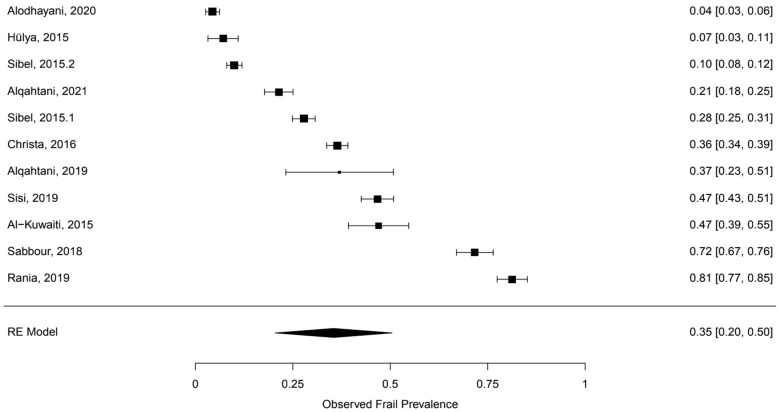
Forest plot of the observed frail prevalence.

A random-effects meta-analysis of 10 studies indicated there was a pooled frailty prevalence rate of 35.4% with a standard error of 0.0765. This pooled prevalence point estimate of 35.4% was statistically significant (*p* < 0.001) with a 95% confidence interval of [20.4, 50.46]. Figure 3 presents the results of the meta-analysis along with upper and lower 95% confidence interval bounds for the pooled prevalence estimates. The test for heterogeneity was found to be significant, Q (df = 10) = 2194.1800, *p* < 0.001. For I2, the I2 of this meta-analysis was found to be 99.58%, which indicated a high heterogeneity between the studies. Regarding the publication bias, both Egger’s regression test and the trim-fill method indicate publication bias is not affecting the results. The results for Egger’s regression test were not statistically significant (z = 0.7875, *p* = 0.4310). The trim-fill method indicated zero studies were missing on either side (Figure 3).

## 4. Discussion

The current research investigated the prevalence of frailty among the elderly in Middle Eastern countries. This systemic review and meta-analysis showed the pooled prevalence of pre-frail and frail in Middle Eastern countries of 39% and 35%, respectively. The reviewed studies revealed a good quality of research in which the results of this systematic review provided important information for healthcare professionals and policymakers in the Middle East.

Compared to results from pooled prevalence of nations by income level, where the high-income and low-to middle-income nations have 8.2% and 12.3%, respectively, pooled prevalence frailty was notably higher at 35.4% (95% CI: 20.4% to 50.4%) [13]. On the other hand, the pooled prevalence of pre-frailty was somewhat lower, as reported by the same study: 39.2% (95% CI: 31.8% to 46.5%) in lower-middle-income, and 43% (95% CI: 35.8% to 49.2%) in upper-middle-income nations. It is worth mentioning that the included studies in the meta-analysis encompass all scales of income, ranging from low to high-income countries. In another study, Da Mata et al. [28] found that the prevalence of frailty was 19.6% across Latin American and Caribbean countries. Our findings were also higher than a recent reported pooled prevalence of frailty of 12% across 62 countries [29]. However, the included studies in this meta-analysis covered a wide age range in the inclusion criteria. Additionally, the lack of a unified assessment tool to diagnose frailty might also explain the variation in prevalence among the included studies.

The study’s findings indicate that the prevalence of frailty is higher in Middle Eastern nations. Despite indications that many of these nations’ populations are rapidly ageing [30], we presently lack information on the incidence of frailty in these populations; this information is essential to health and social care planning. Additionally, consensus on frailty assessment methodologies is necessary to allow for more rigorous comparisons across groups.

Screening of frailty is critical as it can reduce disability, morbidity, mortality, and enhance the quality of life in frail individuals. However, the different methods of screening have different outcomes. Variable frailty assessment tools were used in the reviewed articles which clearly showed significant heterogeneity in the results of frailty prevalence in the Middle East. In addition, the paucity of studies in the Middle East, in general, makes an uncertain assessment of frailty in the Middle East region. More studies are needed examining the net contribution of frailty screening to risk prediction in different settings and populations, and for both clinical and patient-centered outcomes [31].

Ageing, an inevitable process, is commonly measured by chronological age and, as a convention, a person aged 65 years or more is often referred to as ‘elderly’. However, the ageing process is not uniform across the population due to differences in genetics, lifestyle, and overall health [32]. Another interesting finding was observed in the retrieved studies that there was inconsistent cutoff age to define the elderly population which depends on the country and this has affected the prevalence of frailty. For example, prevalence of frailty was higher among those who are older than 65 years which was evident in Al-Kuwaiti, S.J. et al.’s study. This lack of general agreement on a specific age criterion to be used as a consensus standard for increased mortality risk in the elderly results in study heterogeneity, making comparisons between studies difficult [33].

This study has some limitations that should be considered for future research. Although we have used a robust method by including several computerized systematic works of literature, there was limited information on frailty status in other Middle East countries. Therefore, we encourage this area of research to focus on improving the healthcare of older adults in the Middle East. The criteria for measuring frailty in the reviewed studies were based on cut-off scores (Frail, Pre-frail or healthy), dichotomization (Frail or healthy), or phenotype modelling. These criteria might facilitate the heterogeneity of the results, which might need extensive analysis. There was not enough information about the sex differences in the reviewed studies in which future researches are needed to overcome this limitation. Because of the limited number of middle eastern countries that have been included in this systematic review, it is difficult to enhance the generalizability of the results to all countries in the area. Some of the reviewed studies were underpowered or provided unclear information about the recruitment site. Clarifying this information will help in strengthening the methodology and adding a substantial sampling procedure. It has been known the high prevalence of depression, anxiety, and sleep disturbances with ageing might influence the results of frailty. Future research needs to control extraneous variables that might affect the frailty status of older adults.

## 5. Conclusions

From the study findings, the prevalence of frailty might be higher in the included Middle Eastern nations although caution should be applied for interpretations due to limitations in the included studies. Despite indications that many of these nations’ populations are rapidly ageing, in some countries of the middle east, limited data were available, implying the need for greater study in these nations as well as a more systematic method to the design and presentation of prevalence studies.

## Figures and Tables

**Table 1:** Characteristics of studies included in the systematic review.

Author (Year)	Study Country	Sample Size (% Male)	Mean Age	Outcomes	Prevalence of Frailty %	Study Settings	Methods of Sampling	Was the Sample Size Justified? Powered
Al-Kuwaiti et al. (2015) [15]	UAE	160 (53%)	65.6	Fried Frailty Criteria	47	Community-dwelling	Convenience sampling	yes
Alodhayani et al. (2020) [16]	Saudi Arabia	498 (27%)	69.9	Edmonton frailty scale	4.41	Public tertiary hospital	Convenience sampling	yes
Sabbour et al. (2018) [17]	Egypt	350 (50%)	66.3	SHARE Frailty Instrument	71.7	Community + nursing homes	Convenience sampling	no
Sibel et al. (2015) [19]	Turkey	906 (94.4%)	71.5	Fried Frailty Criteria	27.8	Community-dwelling	Random sampling	no
Sibel et al. (2015) [19]	Turkey	906 (94.4%)	71.5	FRAIL scale	10	Community-dwelling	Random sampling	no
Rania et al. (2019) [20]	Iran	555 (52%)	71.5	Tilburg Frailty Indicator	46.7	Community-dwelling	Random sampling	yes
Alqahtani et al. (2021) [23]	Saudi Arabia	486 (65%)	71	Fried Frailty Criteria	21.4	Community-dwelling	Convenience sampling	yes
Alqahtani et al. (2019) [24]	Saudi Arabia	47 (16%)	70	Frail scale	37	Community-dwelling	Convenience sampling	no
Christa et al. (2016) [25]	Lebanon	1200 (46%)	75.7	Study of Osteoporotic Fractures	36.4	Community-dwelling	Random sampling	no
Sisi et al. (2019) [26]	Iran	555 (52%)	71.5	Tilburg Frailty Indicator	46.7	Community-dwelling	Random sampling	yes
Hulya et al. (2015) [27]	Turkey	168 (46%)	72.7	Fried Frailty Criteria	7.1	Community-dwelling	Convenience sampling	yes

**Table 2:** Quality assessment the studies included in the systematic review.

Study	Selection	Comparability	Outcome	Total
Al-Kuwaiti et al. (2015) [15]	***	*	***	7
Alodhayani et al. (2020) [16]	***		**	5
Sabbour et al. (2018) [17]	*		**	3
Sibel et al. (2015) [19]	***	*	***	7
Sibel et al. (2015) [19]	***	*	***	7
Rania et al. (2019) [20]	****		****	8
Alqahtani et al. (2021) [23]	***	*	**	6
Alqahtani et al. (2019) [24]	***		***	6
Christa et al. (2016) [25]	****	*	**	7
Sisi et al. (2019) [26]	****		***	7
Hulya et al. (2015) [27]	***		**	5

From 0–3 stars indicate low-quality, moderate quality (4–6 stars), and high quality (7–9 stars).

## Data Availability

Not applicable.
